# TM6SF2-rs58542926 Genetic Variant Modifies the Protective Effect of a “Prudent” Dietary Pattern on Serum Triglyceride Levels

**DOI:** 10.3390/nu15051112

**Published:** 2023-02-23

**Authors:** Ioanna Panagiota Kalafati, Maria Dimitriou, Konstantinos Revenas, Alexander Kokkinos, Panos Deloukas, George V. Dedoussis

**Affiliations:** 1 Department of Nutrition and Dietetics, School of Health Science and Education, Harokopio University of Athens, 17671 Athens, Greece; 2 Department of Nutrition and Dietetics, School of Physical Education, Sport Science and Dietetics, University of Thessaly, 42100 Trikala, Greece; 3 Department of Nutrition and Dietetics, School of Health Sciences, University of the Peloponnese, Antikalamos, 24100 Kalamata, Greece; 4 Radiology Department, Laiko General Hospital, 111527 Athens, Greece; 5 First Department of Propaedeutic Medicine, School of Medicine, National and Kapodistrian University of Athens, Laiko General Hospital, 11527 Athens, Greece; 6 William Harvey Research Institute, Barts and The London School of Medicine and Dentistry, Queen Mary University of London, London EC1M 6BQ, UK

**Keywords:** TM6SF2, dietary patterns, triglycerides, interaction, NAFLD, genetics, FLI, MBOAT7, PNPLA3, GCKR

## Abstract

The epidemic prevalence of non-alcoholic fatty liver disease (NAFLD), despite extensive research in the field, underlines the importance of focusing on personalized therapeutic approaches. However, nutrigenetic effects on NAFLD are poorly investigated. To this end, we aimed to explore potential gene-dietary pattern interactions in a NAFLD case–control study. The disease was diagnosed with liver ultrasound and blood collection was performed after an overnight fast. Adherence to four a posteriori, data-driven, dietary patterns was used to investigate interactions with PNPLA3-rs738409, TM6SF2-rs58542926, MBOAT7-rs641738, and GCKR-rs738409 in disease and related traits. IBM SPSS Statistics/v21.0 and Plink/v1.07 were used for statistical analyses. The sample consisted of 351 Caucasian individuals. PNPLA3-rs738409 was positively associated with disease odds (OR = 1.575, *p* = 0.012) and GCKR-rs738409 with lnC-reactive protein (CRP) (beta = 0.098, *p* = 0.003) and Fatty Liver Index (FLI) levels (beta = 5.011, *p* = 0.007). The protective effect of a “Prudent” dietary pattern on serum triglyceride (TG) levels in this sample was significantly modified by TM6SF2-rs58542926 (*p*_interaction_ = 0.007). TM6SF2-rs58542926 carriers may not benefit from a diet rich in unsaturated fatty acids and carbohydrates in regard to TG levels, a commonly elevated feature in NAFLD patients.

## 1. Introduction

The wide spectrum of non-alcoholic fatty liver disease (NAFLD) constitutes the most common cause of liver disease worldwide, estimated to currently affect 24% of the global population [1]. Histologically, it ranges from simple steatosis to non-alcoholic steatohepatitis (NASH), and through to advanced fibrosis and cirrhosis [2]. The development of NAFLD is complex and involves the interplay of multiple genetic and environmental factors, including diet [3]. Its epidemic prevalence, coupled with the fact that no drugs have been licensed yet for its treatment, underline the importance of deepening our understanding on NAFLD development to such an extent that an early disease diagnosis, as well as the application of personalized and effective therapeutic approaches, become attainable.

The role of genetics in the pathogenesis of NAFLD is strongly supported by twin and family studies, but also by the diverse prevalence of the disease among different ethnic backgrounds [4]. Its heritability is estimated at 22–50% [5]. To date, large genome-wide association studies (GWAS) have highlighted several single nucleotide polymorphisms (SNPs), which are involved in NAFLD onset and progression through metabolic pathways that mainly involve lipid and glucose metabolism, as well as inflammation [6,7,8,9]. One of the most robustly observed effects, both in lean and obese patients with NAFLD, is located in the PNPLA3 (patatin-like phospholipase domain-containing 3) gene, in which the rs738409 C > G variant is strongly associated with the disease. Other well-replicated NAFLD-associated genetic variants are TM6SF2 (transmembrane 6 superfamily member 2) rs58542926, GCKR (glucokinase regulator) rs780094, and MBOAT7 (membrane-bound O-acyltransferase domain-containing 7) rs641738. 

Diet is a major determinant of NAFLD onset, and nutritional epidemiologists support the study of dietary patterns instead of single nutrients or food groups in studies of chronic diseases [10]. Adherence to dietary patterns, such as Mediterranean diet (MD) and Western diet, has been strongly linked to hepatic steatosis and liver stiffness [11,12,13]. On the other hand, a posteriori assessment of dietary patterns of a population is often used to obtain a better understanding of their habitual dietary habits. Current data on dietary patterns of NAFLD patients are limited [14,15,16,17,18,19]; however, identified patterns are homogenous among different populations.

There is growing evidence that gene-diet interactions play a role in the pathogenesis of chronic metabolic diseases, shedding light on the missing heritability mystery. Hitherto, only a few studies have explored these interactions in NAFLD [20,21,22,23,24,25,26,27]. Since the genetic background of NAFLD patients cannot be modified, it is important to identify favorable dietary factors that could minimize the genetic susceptibility of an individual. Providing patients with personalized dietary and lifestyle advice could reduce NAFLD incidence and improve its management [22]. To this end, the present work attempted to assess potential interactions between known genetic variants and a posteriori, data-driven, dietary patterns in a sample of Greek NAFLD patients and controls.

## 2. Materials and Methods

### 2.1. Study Population

This is a secondary data analysis of a Greek case–control study for NAFLD [20]. Volunteers were consecutively enrolled during the period 2012–2015 from Laiko General Hospital of Athens. Individuals aged 18–65 years old with no self-declared concomitant liver injury at the time of recruitment were screened for NAFLD. Individuals with any congenital or acquired liver disease, chronic viral hepatitis, hepatotoxic drugs exposure, excessive alcohol consumption (more than 20 g of ethanol per day for women and more than 30 g for men), a life-threatening disease or psychiatric disorders impairing the patient’s ability to provide written informed consent, and pregnant or lactating women were excluded from enrolment. The final sample consisted of 351 non-related Caucasian individuals. All study subjects were informed about the aims of the study and signed a written consent. This study was approved by the Ethics Committee of Harokopio University of Athens (38074/13-07-2012), based on the Helsinki Declaration. 

### 2.2. NAFLD Staging and Classification

Participants underwent a liver ultrasound (U/S) in the Radiology Department of the hospital, and all U/S were performed by the same operator to reduce heterogeneity of the results. Staging of NAFLD was diagnosed based on Saadeh S. et al. [28] and were classified into two groups due to similar metabolic and clinical profiles: the control group included individuals with no and mild hepatic steatosis, and cases included moderate and severe hepatic steatosis. This classification allows the depicting of the major contributors to NAFLD development.

### 2.3. Demographic, Clinical, and Anthropometric Data

All participants were interviewed regarding their demographics, family, and individual medical history. Blood collection was performed after a 12 h overnight fast, and blood tests included lipid and glycemic profile, liver enzymes, and uric acid. Low-density lipoprotein cholesterol (LDL-C) was calculated using the Friedewald equation, and the degree of insulin resistance was determined by the homeostatic model assessment (HOMA-IR) [29,30]. Two predictive indices were also calculated in our sample. (i) NFS (NAFLD Fibrosis Score), which constitutes a scoring system validated to separate NAFLD patients with and without advanced fibrosis, and which is calculated based on the following formula: NFS = [−1.675 + 0.037 × age (years) + 0.094 × BMI (kg/m^2^) + 1.13 × IFG/diabetes (yes = 1, no = 0) + 0.99 × AST/ALT ratio − 0.013 × platelet (×109/L) − 0.66 × albumin (g/dL)] [31]. (ii) FLI (Fatty Liver Index), a surrogate index to diagnose fatty liver, which is calculated based on the following formula: FLI = (e^0.953×loge (triglycerides)+0.139×BMI+0.718×loge (gamma-GT)+0.053×waist circumference−15.745^)/(1 + e^0.953×loge (triglycerides)+0.139×BMI+0.718×loge (gamma-GT)+0.053×waist circumference−15.745^) × 100 [32]. Participants underwent anthropometric measurements, and the mean value of two repeated measurements of anthropometric characteristics was reported. Body composition was assessed with an electronic scale (TANITA Segmental Body Composition Analyzer BC-418). Smoking habits were collected and assessed using the following formula: number of pack-years = (number of cigarettes smoked per day/20) × number of years smoked. Physical activity was assessed with the validated short self-reported questionnaire Athens Physical Activity Questionnaire (APAQ) [33]. 

### 2.4. Dietary Assessment and Dietary Patterns Extraction

A 172-food item, semi-quantitative Food Frequency Questionnaire (FFQ) was used to assess the dietary habits of the sample [17]. Factor analysis [Principal components (PCA)] was applied to extract the main dietary patterns of this sample. Four dietary patterns were derived as follows: 1. A “Fast-food-type” pattern, which included fast food, sweetened soft drinks, fried potatoes, and savory and puff pastry snacks. 2. A “Prudent” pattern, which consisted of oil-based cooked vegetables, legumes, potatoes, fruits, vegetables, and fatty fish. 3. A “High-protein” pattern, which included red meat, poultry, and eggs. 4. An “Unsaturated FA” pattern, which included nuts, chocolate, and other foods rich in unsaturated fatty acids. These four factors explained 46% of the sample variability, with a KMO = 0.660 and Bartlett’s test of sphericity <0.001. Adherence of each individual to each of the dietary patterns was classified into four quartiles, where quartile 4 represents the highest adherence to the dietary pattern. Detailed methodology of dietary assessment and dietary patterns generation has been mentioned elsewhere [17]. Misreporting of dietary information was estimated using Goldberg’s method, updated by Black et al. [34,35]. 

### 2.5. DNA Extraction and Genotyping

Buffy coat samples were used to extract DNA, and DNA samples were genotyped using a genome-wide SNP assay (Infinium CoreExome-24 BeadChip, Illumina, San Diego, CA, USA). Genetic information for four SNPs were extracted from the initial database: PNPLA3-rs738409 (C > G), TM6SF2-rs58542926 (G > A), MBOAT7-rs641738 (G > A) and GCKR-rs780094 (G > A). All SNPs satisfied the Hardy–Weinberg equilibrium (HWE), had a minor allele frequency > 5%, and were successfully genotyped in our sample (SNP call rate ≥ 98%). Nine individuals were removed from genetic analyses due to low genotyping rate (sample call rate ≥ 95%). Genetic variants’ information and genotype distribution in NAFLD/control groups and lean/non-lean NAFLD groups are presented in the Supplementary File (Tables S1–S3).

### 2.6. Statistical Analysis

Normality of variables was tested using Kolmogorov–Smirnov test. Mean ± standard deviation (SD) values were used to describe normally distributed quantitative variables, while non-parametric variables were described as median [interquartile range (IQR)]. Relative frequencies (%) were used to describe qualitative variables. To compare differences between the groups, Independent Samples *t*-test (parametric continuous variables) and Mann–Whitney test (non-parametric continuous variables) were performed. Chi-square test was applied to assess dependency of categorical variables. Binary multiple logistic regressions were performed to test the hypothesis of association between various risk factors with the presence of NAFLD. Linear regression models were applied in order to identify associations between risk factors and NAFLD-associated biochemical parameters and indices. Non-parametric continuous variables were log-transformed. Gene–diet interactions were investigated, assuming an additive model for PNPLA3, GCKR, MBOAT7, and a dominant model for TM6SF2. All tests were two-sided and the cut-off level of significance was defined at 0.05. Level of significance for genetic associations was defined as a = 0.0125 after Bonferroni correction. Statistical analyses were performed using IBM SPSS Statistics v21.0 and Plink v1.07.

## 3. Results

Demographic, anthropometric, and clinical characteristics of the study population are displayed in Table 1. NAFLD patients (38% of the sample) had a higher mean age, a lower PAL, and reported less years of education and a higher rate of smoking compared to controls (*p* < 0.05). However, no significant differences were found regarding gender distribution in the sample. As expected, all anthropometric measurements and biochemical parameters were higher in cases compared to controls (*p* < 0.01). Misreporting of dietary intake was not different between the groups (data not shown).

The association of each of the four SNPs with disease odds and disease-related traits was firstly investigated and is presented in Table 2. After adjusting for age, gender, and BMI, PNPLA3-rs738409/G allele was significantly associated with 57.5% higher NAFLD odds compared to C allele (*p* = 0.012). GCKR-rs780094/A was positively associated with lnCRP levels after adjusting for age, gender, BMI, and NAFLD diagnosis (B = 0.098, *p* = 0.003), as well as with higher FLI values after adjusting for age, gender, and NAFLD diagnosis (B = 5.011, *p* = 0.007). The aforementioned associations were not changed after further adjustment for adherence to each dietary pattern (data not shown). No other association reached the corrected *p*-value threshold.

**Figure 1:**
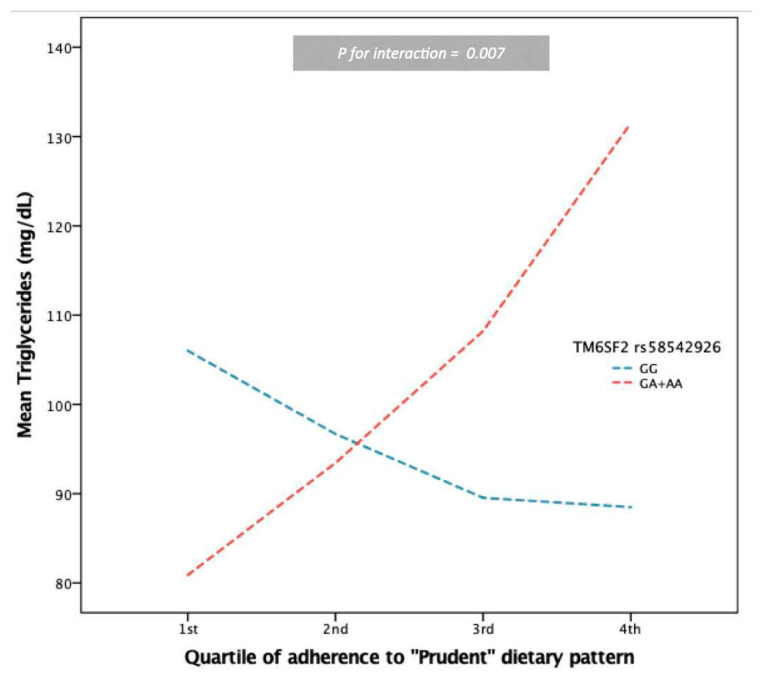
Modification of the protective effect of the “Prudent” dietary pattern on serum triglyceride levels by TM6SF2-rs5854292. Carrying the A allele increased triglycerides by 20.170 mg/dL when adherence to the dietary pattern was higher, compared to non-carriers and after adjusting for age, gender, energy intake, NAFLD diagnosis, adherence to “Prudent” dietary pattern, and genotype (*p*_interaction_ = 0.007). Interaction remained significant after further adjustment for pack-years, education years, and PAL. Bonferroni-corrected significance level threshold: α = 0.0125. Pairwise comparisons indicated significant differences within the 4th quartile of adherence.

Gene-dietary patterns interaction analyses in our sample indicated four associations with a *p*-value ≤ 0.0125 (Table 3). TM6SF2-rs58542926 was found to interact with adherence to the “Prudent” dietary pattern. Carriers of the A allele presented with 20.170 mg/dL elevated triglyceride (TG) levels compared to non-carriers as adherence to this dietary pattern increased, after adjusting for age, gender, energy intake, NAFLD diagnosis, adherence to “Prudent” dietary pattern, TM6SF2-rs58542926, and antilipidemic drug therapy (*p* = 0.007) (Figure 1). In the same context, A carriers had higher FLI levels than non-carriers as adherence increased, after adjusting for age, gender, energy intake, NAFLD diagnosis, adherence to “Prudent” dietary pattern, and TM6SF2-rs58542926 (B = 9.351, *p* = 0.009). On the other hand, carriers of the MBOAT7-rs641738/A allele benefited from a greater adherence to the “Prudent” dietary pattern regarding TG levels (B = −10.06, *p* = 0.003). All interaction results remained significant after further adjustment for pack-years, years of education, and PAL.

## 4. Discussion

Using data from a Greek NAFLD case–control study, the effect of a posteriori dietary patterns on genetic susceptibility to NAFLD and selected NAFLD-related anthropometric and clinical traits was assessed. Results for the sole effect of adherence to four dietary patterns derived with PCA on disease and related traits have been reported in detail elsewhere [17]. In summary, adherence to the “Fast-food-type” pattern was independently associated with higher NAFLD odds, CRP, and uric acid levels (*p* ≤ 0.05), whereas a greater adherence to the “Unsaturated fatty acids” pattern was protective towards NAFLD, insulin, and HOMA-IR levels. The “Fast-food-type” pattern was further associated with higher CRP and uric acid levels and the “Unsaturated fatty acids” pattern with reduced levels of insulin and HOMA-IR (*p* ≤ 0.05). The “Prudent” dietary pattern was associated with decreased TG and uric acid levels (*p* = 0.037 and *p* = 0.035, respectively). As expected, NAFLD patients were older, less educated, less active, and presented with worse anthropometric and biochemical background than controls. NFS and FLI scores were higher in the patients group; however, mean NFS values are <−1.455, which indicates that this is a group of NAFLD patients without fibrosis, on average.

Herein, the well-known positive association of rs738409-G with NAFLD odds was replicated. This genetic variant is known to be robustly associated with hepatic fat accumulation and elevated liver enzymes [36]. However, no significant associations were observed as regards liver enzymes’ levels, possibly due to the fact that this sample most probably consists of simple fatty liver patients rather than patients with advanced NASH and fibrosis (as assessed through NFS results and mean liver enzymes which are within normal range). Carrying the GCKR-rs780094/A allele was associated with higher lnCRP and FLI levels. The effect of the glucose-lowering allele A on CRP levels has been previously reported in various populations [37,38,39,40]. CRP is the prototype acute-phase reactant and a hallmark of low-grade systemic inflammation, thus it has been assumed that chronic low-grade systemic inflammation mediates the effect of the GCKR variant on complex metabolic diseases, including NAFLD.

No association results reached the Bonferroni-corrected *p*-value threshold for TM6SF2 and MBOAT7 variants. The lack of evidence of association of TM6SF2 could be explained by the low effect allele frequency (EAF) of the SNP in our sample (EAF = 5.6%), highlighting the need for a larger sample size. A dominant genetic model was assumed for this SNP. Moreover, the fact that this is mainly a non-fibrotic NAFLD group of patients could explain the lack of association of r641738; the latter has been mainly linked to advanced disease stages through altering the remodeling of phosphatidylinositol [41,42]

In this present study, two significant rs58542926-“Prudent” dietary pattern interactions were reported. Carriers of the A allele who adhered better to the pattern had higher TG values than non-carriers, independent of age, gender, energy intake, NAFLD diagnosis, adherence to “Prudent” dietary pattern, TM6SF2-rs58542926 and antilipidemic drug therapy. In line with this, carrying at least one copy of the A allele significantly interacted with adherence to the pattern to affect FLI levels, meaning that these individuals had greater odds of having NAFLD. However, since in this study no significant effects of rs58542926 or the dietary pattern on FLI levels were found, this interaction could potentially be mediated by the aforementioned interaction, since TG levels constitute a component of the FLI.

Carriers of rs58542926, a missense polymorphism, have been found to experience higher levels of intrahepatic fat content and, thus, higher odds of developing NAFLD. The altered protein is believed to modify the excretion of TG and lipid droplets content from the liver, implying a lowering effect of the risk allele of rs58542926 on serum TG levels [43,44,45]. Hypertriglyceridemia constitutes a common feature and a major risk factor for NAFLD onset and progression [46]. In this study no significant effect of the polymorphism on TG levels was found. Nevertheless, given the fact that the association of the dietary pattern with TG levels and the interaction association had opposite directions, this is a valuable result that should be further investigated. Moreover, as Wang et al. suggested, this gene–environment interaction may be a “pure interaction”, meaning that the effect of one exposure is present only in the presence of the other [47]. 

Notably, previously published results from the same sample had indicated a significant interaction between the TM6SF2 variant and fish/fatty fish intake on TG levels [20]. Fish, and especially fatty fish, constitute a rich in polyunsaturated fatty acids (PUFA) food group [20]. At the same time, TM6SF2 protein is believed to constitute a novel key regulator of postprandial lipemia [45,48,49]. Malfunction of this protein may result in impaired lipids integration into TG, leading to increased lipid accumulation and decreased lipid export, which as a result leads to lower serum TGs levels. Indeed, in their study Musso et al. indicated that TM6SF2-A carriers experienced lower postprandial TG levels, along with lower non-esterified fatty acids and oxLDL responses and higher HDL-C levels, after an oral fat tolerance test [50]. However, in their RCT, Scorletti et al. reported no significant influence of the TM6SF2 genotype on the effect of DHA + EPA supplementation of NAFLD patients [51]. Taken together, it was hypothesized that in response to high PUFA intake, carriers of the TM6SF2 variant experience increased intrahepatic accumulation of TGs and thus hepatic steatosis. 

In line with the above, we could assume a similar effect of rs58542926 on individuals with greater adherence to the “Prudent” dietary pattern. The latter constitutes (a) a combination of two rich sources of fatty acids, namely the olive oil (included in oil-based cooked vegetables) and the fatty fish, along with (b) carbohydrate- and fructose-rich food groups, namely fruits, potatoes, legumes, and vegetables. High sugars and especially fructose intake have been associated with greater odds for NAFLD [52]. There is also evidence that TM6SF2 may be implicated in glucose metabolism. Lei et al. reported that TM6SF2 protein up-regulation is mediated by carbohydrate-responsive element-binding protein (ChREBP) [53], which in turn is stimulated by fructose intake. Moreover, it was recently shown that rs58542926 is associated with glucose intolerance both in humans and mice [54]. It could thus be hypothesized that G-allele carriers are susceptible to a diet combining high unsaturated fat and high carbohydrate foods as regards TG levels. However, the current scientific literature lacks data regarding interactions of TM6SF2 with distinct dietary patterns or food groups. More research is required to validate the nutritional regulation of TM6SF2, and large-scale RCTs as well are needed in order to clarify the potential interaction of PUFAs/total fat/carbohydrate intake and TM6SF2 polymorphism in NAFLD and its related traits.

Unlike TM6SF2, carriers of the MBOAT7-rs641738/A allele benefited from a greater adherence to the “Prudent” dietary pattern regarding TG levels (*p* = 0.003). Due to the lack of direct association of the genetic variant with TG levels, along with the same direction of the interaction effect, it could be assumed that this interaction reflects the effect of the dietary pattern on serum TG levels. 

This study has some limitations that should be considered. The size of our sample is modestly small, which limits the probability of detecting genetic and nutrigenetic associations. Moreover, retrospective case–control studies, especially those conducted in a clinical setting, are potentially prone to analysis and sampling bias. Herein, NAFLD diagnosis was based on liver U/S, a method greatly dependent on the operator with 60–94% sensitivity and 84–95% specificity for detecting fatty liver. Last but not least, despite all actions taken to eliminate it, self-report of dietary intake is susceptible to recall bias.

## 5. Conclusions

Despite its limitations, this is a novel study investigating for the first time the interplay between four genetic variants and four dietary patterns in NAFLD. We showed that rs58542926 in the TM6SF2 locus is a potential modifier of the protective effect of a “Prudent” dietary pattern on serum TG levels. The area of gene–diet interactions constitutes a large and diverse puzzle, and although it is not easy to solve, its gradual deciphering may take NAFLD management to the next level. To this end, large-scale RCTs and prospective studies aiming at investigating gene–diet interactions in NAFLD are very much needed.

## Figures and Tables

**Table 1:** Demographic, anthropometric, and clinical characteristics of the study population. All individuals were of Caucasian ancestry.

	Controls (*n* = 217)	Cases (*n* = 134)	*p*-Value
Gender (% males)	39.2	45.5	0.144
Age (years)	43.75 ± 11.23	50.36 ± 10.51	<0.001
Education years	15.25 ± 3.60	14.02 ± 3.99	0.005
PAL	1.43 ± 0.22	1.38 ± 0.23	0.011
Pack-years	8.08 ± 13.71	15.19 ± 24.49	0.024
BMI (kg/m^2^)	24.92 ± 3.27	31.11 ± 4.72	<0.001
WHR	0.83 ± 0.09	0.92 ± 0.08	<0.001
Fat Mass (%)	26.23 ± 8.64	33.76 ± 8.93	<0.001
ALT (U/L)	21.44 ± 11.73	30.26 ± 14.51	<0.001
AST (U/L)	21.10 ± 6.77	23.75 ± 8.44	0.002
γ-GT (U/L)	19.98 ± 17.58	28.29 ± 21.72	<0.001
TC (mg/dL)	195.44 ± 38.85	209.19 ± 33.66	0.001
LDL (mg/dL)	120.99 ± 33.05	132.77 ± 30.38	0.001
HDL (mg/dL)	57.87 ± 14.42	50.98 ± 12.42	<0.001
TG (mg/dL)	78.51 ± 37.31	127.43 ± 62.64	<0.001
FGlu (mg/dL)	84.39 ± 8.27	93.33 ± 12.65	<0.001
FIns (µU/mL)	9.09 (4.30)	13.40 (7.81)	<0.001
HbA1c (%)	5.23 ± 0.34	5.56 ± 0.44	<0.001
HOMA-IR	1.84 (1.84)	3.06 (2.22)	<0.001
CRP (mg/L)	2.30 (4.30)	2.34 (1.84)	<0.001
Uric Acid (mg/dL)	4.73 ± 1.17	5.65 ± 1.25	<0.001
NFS	−2.76 ± 0.93	−1.99 ± 1.26	<0.001
FLI	21.47 ± 20.23	64.78 ± 24.37	<0.001

Values given as mean ± SD/median (IQR) for quantitative variables and relative frequencies (%) for categorical variables. *p*-value: *t*-test/Mann–Whitney *p*-value for quantitative and chi-square *p*-value for categorical variables. PAL: Physical activity level; BMI: Body mass index; WHR: Waist-to-hip ratio; ALT: Alanine transaminase; AST: Aspartate transaminase; γ-GT: Gamma-glutamyltransferase; TC: Total cholesterol; LDL: Low-density lipoprotein; HDL: High-density lipoprotein; TG: Triglycerides; FGlu: Fasting glucose; FIns: Fasting insulin; HbA1c: Hemoglobin A1c; HOMA-IR: homeostat.ic model assessment; CRP: C-reactive protein; NFS: NAFLD fibrosis score; FLI: Fatty Liver Index.

**Table 2:** Genetic associations with NAFLD and disease-related traits.

	PNPLA3-rs738409/G			TM6SF2-rs58542926/A			GCKR-rs780094/A			MBOAT7-rs641738/A		
	**OR**	**95% CI**	***P* ^†^**	**OR**	**95% CI**	***P* ^†^**	**OR**	**95% CI**	***P* ^†^**	**OR**	**95% CI**	***P* ^†^**
NAFLD *	1.575	1.104–2.245	***0.012***	1.482	0.710–3.092	0.294	1.108	0.775–1.584	0.574	1.147	0.829–1.588	0.407
	**Beta**	**SE**	***P* ^†^**	**Beta**	**SE**	***P* ^†^**	**Beta**	**SE**	***P* ^†^**	**Beta**	**SE**	***P* ^†^**
WHR	−0.005	0.007	0.505	0.015	0.015	0.336	0.011	0.007	0.129	−0.002	0.007	0.791
Fat mass (%)	0.084	0.680	0.902	0.715	1.44	0.620	0.833	0.668	0.213	−0.734	0.624	0.241
ALT (U/L)	1.712	1.071	0.111	−0.066	2.281	0.977	1.216	1.063	0.253	0.714	0.987	0.470
AST (U/L)	0.215	0.6186	0.728	−0.250	1.313	0.850	1.377	0.614	0.026	0.041	0.569	0.943
γ-GT (U/L)	−0.470	1.660	0.778	8.290	3.520	0.019	2.575	1.635	0.116	−1.116	1.513	0.461
TC (mg/dL) ^§^	5.043	3.984	0.207	−8.089	6.42	0.209	−1.046	5.509	0.850	6.536	4.277	0.128
HDL (mg/dL) ^§^	2.077	1.110	0.062	−2.859	2.359	0.226	1.479	1.109	0.184	1.442	1.021	0.159
LDL (mg/dL) ^§^	2.326	2.574	0.376	−6.525	5.449	0.232	0.983	2.581	0.704	2.908	−1.717	0.219
TG (mg/dL) ^§^	−5.504	4.036	0.174	0.334	8.581	0.969	−0.442	4.047	0.913	−3.126	3.712	0.400
FGlu (mg/dL) ^¶^	−0.617	0.794	0.438	1.483	1.651	0.369	0.407	0.779	0.602	0.541	0.715	0.450
lnFIns (µU/mL) ^¶^	−0.045	0.036	0.214	−0.040	0.076	0.597	−0.002	0.036	0.945	0.004	0.033	0.910
HbA1C (%) ^¶^	0.068	0.038	0.072	−0.076	0.090	0.400	0.039	0.037	0.296	−0.010	0.033	0.764
lnHOMA-IR ^¶^	−0.037	0.040	0.360	−0.029	0.084	0.727	0.010	0.039	0.794	0.016	0.036	0.665
lnCRP (mg/L)	−0.024	0.034	0.484	−0.022	0.073	0.322	0.098	0.033	***0.003***	−0.005	0.030	0.873
Uric Acid (mg/dL)	−0.227	0.103	0.029	0.179	0.228	0.433	0.075	0.102	0.464	−0.147	0.093	0.116
NFS **	0.016	0.091	0.859	0.244	0.193	0.207	−0.038	0.090	0.673	−0.049	0.083	0.552
FLI ***	−3.969	1.871	0.035	0.950	3.998	0.812	5.011	1.840	***0.007***	−2.848	1.689	0.093

Multiple logistic and linear regression models for the association of selected SNPs in PNPLA3, TM6SF2, GCKR, and MBOAT7 genetic loci with NAFLD. An additive model of inheritance was assumed for all variants, except TM6SF2-rs58542926 where analysis was based on a dominant model. Age, gender, BMI, and NAFLD diagnosis were used as confounding factors in the models. Significant results have been marked in bold and italics. OR: Odds ratio; 95% CI: 95% confidence interval; SE: Standard error; WHR: Waist-to-hip ratio; ALT: Alanine transaminase; AST: Aspartate transaminase; γ-GT: Gamma-glutamyltransferase; TC: Total cholesterol; LDL: Low-density lipoprotein; HDL: High-density lipoprotein; TG: Triglycerides; FGlu: Fasting glucose; FIns: Fasting insulin; HbA1c: Hemoglobin A1c; HOMA-IR: homeostatic model assessment; CRP: C-reactive protein; NFS: NAFLD fibrosis score; FLI: Fatty Liver Index. * Model adjusted for age, gender and bmi. ** Model adjusted for gender and NAFLD diagnosis. *** Model adjusted for age, gender, and NAFLD diagnosis. ^§^ Model additionally adjusted for antilipidemic drug therapy. ^¶^ Model additionally adjusted for antidiabetic drug therapy. ^†^ Bonferroni-corrected significance level threshold: α = 0.0125.

**Table 3:** Significant interactions of adherence to the “Prudent” dietary pattern with genetic variants.

	Model 1 *			Model 2 **		
	Beta_interaction_	SE_interaction_	*P*_interaction_^†^	Beta_interaction_	SE_interaction_	*P*_interaction_^†^
TM6SF2-rs58542926/A * “Prudent” Dietary Pattern						
TG (mg/dL) ^§^	20.170	7.435	0.007	20.530	7.262	0.005
FLI	9.351	3.545	0.009	9.568	3.503	0.007
MBOAT7-rs641738/A * “Prudent” Dietary Pattern						
TG (mg/dL) ^§^	−10.060	3.400	0.003	−9.993	3.375	0.003

Multiple linear regression models with interactions of TM6SF2 and MBOAT7 genetic variants with adherence to “Prudent” dietary pattern. An additive model of inheritance was assumed for MBOAT7-rs641738 and a dominant model for TM6SF2-rs58542926. Only results that reached the corrected significance level are shown in the table. SE: Standard error; TG: Triglycerides; FLI: Fatty Liver Index. * Model 1 is adjusted for age, gender, energy intake, NAFLD diagnosis, adherence to “Prudent” dietary pattern, and genotype. ** Model 2 is adjusted for age, gender, energy intake, NAFLD diagnosis, adherence to “Prudent” dietary pattern, genotype, pack-years, education years, and PAL. ^§^ Model additionally adjusted for antilipidemic drug therapy. ^†^ Bonferroni-corrected significance level threshold: α = 0.0125.

## Data Availability

The data presented in this study are available on request from the corresponding author. The data are not publicly available due to their containing information that could compromise the privacy of research participants.

## Supplementary Materials

The following supporting information can be downloaded at: https://www.mdpi.com/article/10.3390/nu15051112/s1, Table S1: Genetic variants information in the sample; Table S2: Genotype distribution in NAFLD patients and controls; Table S3: Genotype distribution in lean and non-lean (overweight/obese) NAFLD patients.
