# Supplementary material for: TM6SF2-rs58542926 Genetic Variant Modifies the Protective Effect of a “Prudent” Dietary Pattern on Serum Triglyceride Levels
Source: Nutrients. 2023 Feb 23;15(5):1112. doi: 10.3390/nu15051112 (PMC10005630; doi:10.3390/nu15051112)
Supplement: Supplementary file 1 [file nutrients-15-01112-s001.zip › nutrients-2216618-supplementary.pdf]

Table S1. Genetic variants information in the sample

SNP	Gene	Chr	Strand	Build	Position	Effect Allele	Non Effect Allele	HWE p-value	EAF
rs738409	PNPLA3	22	+	37	44324727	G	C	0.292	0.290
rs58542926	TM6SF2	19	-	37	19379549	A	G	1	0.056
rs780094	GCKR	2	+	37	27741237	A	G	0.096	0.573
rs641738	MBOAT7	19	+	37	54173068	A	G	0.510	0.445

SNP: Single nucleotide polymorphism; Chr: Chromosome; HWE p-value: Hardy-Weinberg Equilibrium test p-value; EAF: Effect allele frequency

Table S2. Genotype distribution in NAFLD patients and controls

	Controls (N=213)	Cases (N=129)	p-value
PNPLA3 - rs738409 (%)			
CC	54.0	48.1	0.040
CG	39.4	37.2	
GG	6.6	14.7	
TM6SF2 - rs58542926 (%)			
GG	91.5	85.3	0.073
GA/AA	8.5	14.7	
GCKR - rs780094 (%)			
GG	14.6	17.8	0.435
GA	56.1	49.6	
AA	29.2	32.6	
MBOAT7 - rs641738 (%)			
GG	32.9	31.0	0.859
GA	46.9	47.3	
AA	20.2	21.7	

p-value: Chi-Square test p-value

Table S3. Genotype distribution in lean and non-lean (overweight/obese) NAFLD patients

	Lean NAFLD (N=6)	Non-lean NAFLD (N=123)	p-value
PNPLA3 - rs738409 (%)			
CC	16.7	49.6	0.218
CG	50.0	36.6	
GG	33.3	13.8	
TM6SF2 - rs58542926 (%)			
GG	66.7	86.2	0.215
GA/AA	33.3	13.8	
GCKR - rs780094 (%)			
GG	33.3	17.1	0.513
GA	50.0	49.6	
AA	16.7	33.3	
MBOAT7 - rs641738 (%)			
GG	16.7	31.7	0.609
GA	66.7	46.3	
AA	16.7	22.0	

p-value: Chi-Square test p-value
